# Cell-Penetrating Peptide as a Means of Directing the Differentiation of Induced Pluripotent Stem Cells

**DOI:** 10.3390/ijms161125986

**Published:** 2015-11-06

**Authors:** Taku Kaitsuka, Kazuhito Tomizawa

**Affiliations:** Department of Molecular Physiology, Faculty of Life Sciences, Kumamoto University, 1-1-1 Honjyo, Kumamoto 860-8556, Japan; kaitsuka@kumamoto-u.ac.jp

**Keywords:** cell-penetrating peptide, poly-arginine, protein transduction, induced pluripotent stem cell, pancreatic differentiation

## Abstract

Protein transduction using cell-penetrating peptides (CPPs) is useful for the delivery of large protein molecules, including some transcription factors. This method is safer than gene transfection methods with a viral vector because there is no risk of genomic integration of the exogenous DNA. Recently, this method was reported as a means for the induction of induced pluripotent stem (iPS) cells, directing the differentiation into specific cell types and supporting gene editing/correction. Furthermore, we developed a direct differentiation method to obtain a pancreatic lineage from mouse and human pluripotent stem cells via the protein transduction of three transcription factors, Pdx1, NeuroD, and MafA. Here, we discuss the possibility of using CPPs as a means of directing the differentiation of iPS cells and other stem cell technologies.

## 1. Introduction

**Figure 1:**
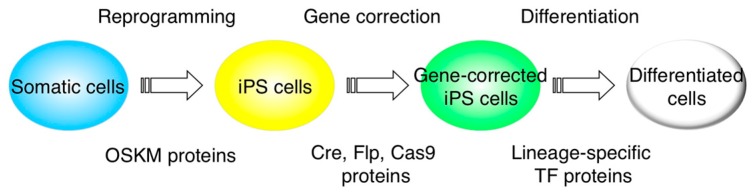
CPP-mediated protein transduction technologies in reprogramming, gene editing/correction, and differentiation of iPS cells. CPP-mediated protein transduction methods are used for key steps in iPS cell technologies. The reprogramming of somatic cells is induced with Yamanaka-4 factors fused to CPPs. Gene correction of disease-specific mutation is performed by the CRISPR-Cas9 system with CPP-fused Cas9 endonuclease. The differentiation of iPS cells is directed with CPP-fused transcription factors. OSKM, Oct4, Sox2, Klf4, c-Myc; TF, transcription factor.

Induced pluripotent stem (iPS) cells are generated from somatic cells and they have a capacity to differentiate into multiple cell types [1]. The use of iPS cell technologies in regenerative medicine involves the key steps of reprogramming, gene editing/correction, and differentiation (Figure 1). Protein transduction via cell-penetrating peptides (CPPs) is a method for the delivery of peptides, recombinant proteins, and large molecules [2]. This method is safer than gene delivery via viral vectors because there is no risk of the genomic integration of exogenous genes. Therefore, this method has the possibility to substitute for virus-mediated gene delivery in the multi steps of reprogramming, gene editing/correction and differentiation (Figure 1). In this review, we summarize recent reports in this field and the future possibility of utilizing this method in iPS cell technologies.

## 2. CPP-Mediated Protein Transduction

It has been hypothesized that eukaryotic cells gained the function of endocytosis via evolution from a common origin of prokaryota [3]. Endocytosis was essential for biological diversity via the acquisition of mitochondria in animals and chloroplasts in plants [3]. Proteins fused with CPPs are internalized into cells via macropinocytosis [4,5], which is a form of fluid phase endocytosis [6]. Cell types with a macropinocytosis process can be transduced with recombinant proteins via CPPs. The CPP sequence was originally found in natural proteins as the HIV trans-activator of transcription (TAT) [7,8] and the Drosophila melanogastor homeodomain transcription factor Antennapedia [9]. That sequence in these proteins with the capacity of penetrating cells is called the protein transduction domain (PTD). Both TAT and Antennapedia contain arginine and lysine-rich residues in their PTDs [2]. Recombinant proteins fused to their PTD sequences or artificial CPPs like arginine-rich peptide (poly-arginine) can internalize into cells. In general, 6 to 12 arginines exhibit transduction activity as CPPs [10,11], while it has recently been reported that three arginines are sufficient for transduction capacity [12]. The first step of protein internalization into cells is mediated via binding to heparan sulfate proteoglycans, recruiting activated GTPase Rac1 to lipid rafts, followed by macropinocytosis [4,13,14,15,16]. However, there are some reports showing that heparan sulfate proteoglycans are not necessary for protein transduction [17,18,19]; therefore, detailed mechanisms are largely unknown. Several molecules including Rac1, p21-activated kinase 1 (Pak1), phosphatidylinositol 3-kinase, oncogene Ras, Src, histone deacetylase 6 (Hdac6), and heat shock protein 90 (Hsp90) have been implicated in macropinocytosis [20], suggesting that these molecules could influence the efficiency of protein transduction. Furthermore, it has been reported that protein entry into cells is also regulated by various molecules, such as coatomer subunit alpha and Na^+^/HCO_3_^−^ cotransporter [21]. Recently, a unique method was reported, involving the intracellular delivery of naïve protein (not fused to any CPPs) via NaCl hypertonicity-induced macropinocytosis and a transduction compound, propanebetaine [22]. Surprisingly, the authors found these components in the buffer used on the purification of recombinant proteins. They also found that Na^+^/H^+^ exchanger 1 (Nhe1) plays an important role in this hypertonicity-induced protein transduction. Furthermore, another group also showed a transduction method without CPPs, involving the cationic lipid-mediated delivery of proteins with negatively supercharged proteins [23]. They used commonly available cationic lipid nucleic acid transfection reagents, lipofectamines.

## 3. Protein Transduction into iPS Cells

**Figure 2:**
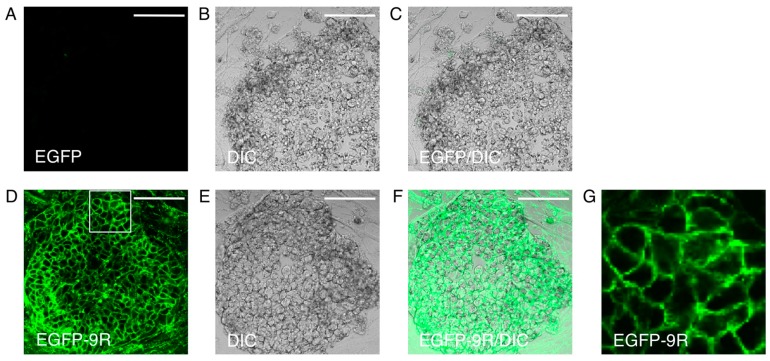
Protein transduction into human iPS cells. Human iPS cells of 201B7 were treated with EGFP or 9R-EGFP for 6 h at a final concentration of 1 µM and GFP fluorescence was analyzed by confocal microscopy. (**A**–**C**) EGFP-treated cells. Images of EGFP fluorescence (**A**); DIC (**B**) and their merge (**C**) were shown. (**D**–**F**) EGFP-9R-treated cells. Images of EGFP fluorescence (**D**); DIC (**E**) and their merge (**F**) were shown; (**G**) Magnified image of indicated area by white box in (**D**). GFP fluorescence was detected in the cytoplasm and cell membrane. Scale bars are 100 µm. 9R, nine arginines. DIC, differential interference contrast.

In general, lentivirus or retrovirus is used as a carrier for exogenous gene transduction to express protein, knockdown, or to edit endogenous genes in iPS cells and embryonic stem (ES) cells. These methods show high transduction efficiency; however, they lead to the integration of exogenous DNA into chromosomes of host cells, especially when viral vectors are used [24]. In the case of gene editing, the random occurrence of a deleterious mutation cannot be ruled out. Plasmid DNA transfection with cationic lipids can reduce the risk of integration into chromosomes; however, almost all pluripotent stem cells are generally difficult to transfect and the transfection efficiency is relatively low. Electroporation is a robust method to increase the efficiency of transfection, but it often leads to cell injury and death. The transduction methods combined with *piggyBac* transposon were developed for iPS cell generation as minimized genomic integration and the complete elimination of exogenous reprogramming factors, for application to regenerative medicine [25,26]. DNA transposons are genetic elements that can relocate between genomic sites by a “cut and paste” mechanism. Important features of the *piggyBac* transposon is that it transposes efficiently in many different species and that it nearly always excises itself precisely and leaves no footprint behind [27,28]. The *piggyBac* system has been shown to be applicable to human and mouse cell lines and this system becomes very attractive as a genetic tool. This *piggyBac* system has recently attracted attention, such as for the reprogramming of somatic cells and purification of differentiated cells [29]. A protein transduction method could also be useful for the transduction of exogenous proteins into iPS cells because of their high transduction efficiency and zero risk of genomic integration. In fact, proteins fused to poly-arginine were efficiently transduced into human iPS cells, whereas proteins without CPPs were not (Figure 2; unpublished data) [30]. In these cells, the signals of transduced EGFP-9R proteins were detected in the cytoplasm and cell membrane.

Macropinocytosis occurs in most cell types, including pluripotent stem cells. Endocytosis processes are important in pluripotent stem cells for nutrient absorption [31], cellular signaling like Notch [32], Wnt [33,34], and gap junctional intercellular communication [35]. Under extracellular stimulation, GTPase, Rac1, and Cdc42 activate Pak1 [36] and these proteins trigger the active rearrangement of the actin cytoskelton and lead to macropinocytosis [20]. ES cells have been reported to express Rac1 and Cdc42, which regulate their migration [37]. In cancer cells, macropinocytosis is stimulated by the oncogene Ras, being important for macropinocytosis [38,39]. ES cells express embryonic stem cell-expressed Ras (E-Ras) [40], which have function in macropinocytosis in ES and iPS cells; however, its role is relatively unknown. Molecular mechanisms of macropinocytosis in iPS cells have also attracted research interest in this field.

## 4. iPS Cell Differentiation with Protein Transduction of Specific Transcription Factor

**Figure 3:**
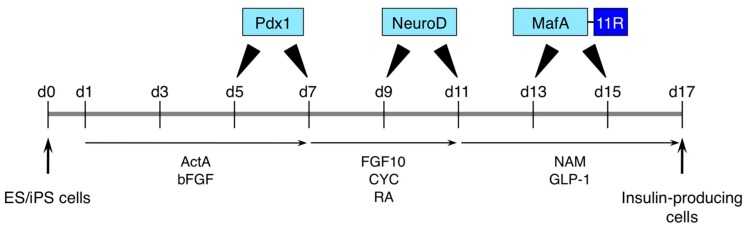
Scheme of the protocol for pancreatic differentiation with Pdx1, NeuroD and MafA-11R protein transduction. Dissociated mouse ES or iPS cells were plated at day 0 and directed to pancreatic differentiation in medium supplemented with Activin A (ActA) and basic fibroblast growth factor (bFGF) from days 1 to 7, followed by medium supplemented with fibroblast growth factor 10 (FGF10), KAAD-cyclopamine (CYC), and retinoic acid (RA) from day 7 to 11, and medium supplemented with nicotinamide (NAM) and glucagon-like peptide-1 (GLP-1) from day 11 to 17. At day 17, a part of differentiated cells express insulin and mature pancreatic β-cell markers. Blue boxes show recombinant proteins of Pdx1, NeuroD, and MafA-11R and these proteins were added at the indicated time-points. d: day; 11R: 11 arginine.

In the general method, some cytokines and growth factors are used to mimic organ development in pluripotent stem cells and direct the differentiation into specific cell types. Small molecules are also used to inhibit selective molecular signaling and guide to specific molecular activation. There have been several reports of efficient methods promoting differentiation from human iPS cells into neurons [41], retinal cells [42], lung cells [43], and pancreatic β cells [44], using some cytokines, growth factors, and small molecules. In addition to these biological factors and chemicals, the protein transduction of specific transcription factors is a useful method for directing the differentiation. We previously developed a differentiation method by the step-wise transduction of recombinant Pdx1, NeuroD, and MafA-11R proteins [45]. Pdx1 and NeuroD have their own PTDs [46,47], while MafA is fused with 11 arginines (11R) as CPPs. In mouse ES cells, these three proteins improved the efficiency of differentiation into insulin-producing cells (Figure 3) [45]. In human iPS cells, culture in differentiation medium with recombinant Pdx1 facilitates differentiation into pancreatic endocrine progenitors [45]. In this method, the order of transduced proteins and timing of protein transduction are important components for efficient differentiation. The protocol of Pdx1 transduced on days 5 and 7, NeuroD on days 9 and 11, and MafA-11R on days 13 and 15, was the most efficient by determining the number of insulin-positive cells, while a different order and timing reduced this efficiency (Figure 3) [45]. The order of transduced proteins is similar to developmental expression *in vivo*. Thus, the order of Pdx1, NeuroD, and MafA-11R is crucial for differentiation into insulin-producing cells. However, the yield of insulin-producing cells was relatively low (~1%). One reason is that full activity of protein transduced into cells was not achieved with this simple method using CPP-containing proteins. A well-optimized protocol for protein transduction will be required. Recently, well-established protocols for the pancreatic differentiation of human iPS cells have been reported [44,48,49]. These protocols use many cytokines, growth factors, hormones, and chemicals. Molecules originating from endogenous factors are thought to be safer and suited for usage in regenerative medicine, although some chemicals have a risk of mutagenicity. In these protocols, toxic chemicals are used, such as phorbol dibutyrate [44]. The possibility that differentiated cells cause tumorigenesis cannot be ruled out. It will be necessary to replace mutagenic chemicals with safe materials as recombinant proteins.

Specific transcription factors are used for directing differentiation into other cell types. For neural differentiation, the forced expression of Ngn2 by lentiviral vectors is used for the efficient induction of functional neurons [50]. Transient overexpression of Nkx2-1 and Pax8 directs the differentiation of mouse ES cells into thyroid follicular cells [51]. Mesp1 expression in the doxycycline-inducible Mesp1 ES cell line promotes skeletal myogenic derivates in the absence of serum-derived factors [52] and the inducible expression of MyoD by *piggyBac* vector leads to efficient differentiation into mature myocytes [53]. In pancreatic differentiation, the combined expression of Pdx1 and MafA with either Ngn3 or NeuroD by adenoviral vectors facilitates the differentiation of mouse ES cells into insulin-producing cells [54]. Each method uses exogenous genes for the induction of transcription factors with lentiviral, adenoviral, and *piggyBac* vectors. These transduction methods cannot exclude the risk of the genomic integration of exogenous DNA and such methods are not desirable for clinical application. The protein transduction method is safer than viral vectors because there is no risk of genomic integration. Therefore, this method has the capacity to substitute for such transcription factors. Recently, there have been several reports of a differentiation protocol with protein transduction, as neural induction by Nkx2.2, Olig2, or Pax6 [55,56,57], myogenic induction by MyoD [58,59]. It is hoped that this method will become widely used for directing the differentiation.

## 5. Gene Editing with CPP-Mediated Protein Transduction

The protein transduction method via CPPs is also useful for introducing Cre recombinase and FLP recombinase proteins into cells to excise target genes [60,61,62] and for introducing Cas9 endonuclease and guide RNA to edit or correct genes [63]. Recently, D’Astolfo’s group and Zuris’s group reported native protein transduction via the hypertonicity- or cationic lipid-mediated delivery of Cre and Cas9, respectively [22,23] and D’Astolfo’s group also succeeded in Cas9 protein transduction into H1 human ES cells by this method [22]. Furthermore, protein transduction via CPPs can be used for siRNA delivery into pluripotent stem cells by fusing siRNA to the RNA-binding domain with CPPs [64]. These technologies are now being used in human pluripotent stem cells as a research element, especially TAT-Cre-mediated gene excision [65,66,67]. Gene-editing/correction technologies in iPS cells are desired for generating disease models carrying specific mutations or the transplantation of gene-corrected autologous tissues [68,69]. Thus the protein transduction method is also attractive in this gene-editing technology as a method without exogenous genes.

## 6. Usage of Protein Transduction in iPS Cell Generation or Direct Conversion

In contrast to directing the differentiation of stem cells, there is some difficulty in reprogramming somatic cells to iPS cells and the direct conversion of somatic cells to other cell types with protein transduction. Some groups reported the generation of mouse or human iPS cells by protein transduction via CPPs [70,71,72]. We have also attempted the reprogramming of fibroblasts with protein transduction of Oct4, Sox2, Klf4, and c-Myc proteins. However we failed to generate iPS cell colonies. In previous reports, the efficiency of iPS cell generation by proteins was significantly lower (about 0.001%) [71] compared to transduction via retroviral vectors (0.02%) [1]. Furthermore, it was reported that cells transduced with these four proteins via CPPs resembled the ES cell morphology but failed to expand like iPS cells; therefore, only partial reprogramming occurred using this method [73]. For complete reprogramming, the robust expression of the four factors might be needed equally to retroviral vector-mediated transduction. To use this for clinical utilization, more efficient protocols with robust expression are needed for this protein-mediated reprogramming.

Direct conversion occurs by the robust expression of specific transcription factors. Ascl1, Brn2, and Myt1l convert fibroblasts into neurons [74], Gata4, Mef2c, and Tbx5 convert fibroblasts into cardiomyocytes [75], Gata4, Hnf1α, and Foxa3 and the inactivation of p19Arf convert fibroblasts into hepatocytes [76], Hnf4α plus Foxa1, Foxa2, and Foxa3 convert fibroblasts into hepatocytes [77] and Sox10, Olig2, and Zfp536 convert fibroblasts into oligodendrocyte precursor cells [78]. They used retroviral or lentiviral vectors for gene transduction and the robust expression of these transcription factors. The protein transduction method has the capacity to replace these viral vector-mediated transductions; however, there is no report at present. Practical protocols are desired regarding protein-mediated direct conversion.

## 7. Conclusions

As stated above, it has been shown by many reports that some steps in iPS cell technologies can be done by protein transduction methods (Table 1). The transduction of exogenous genes via plasmids, viral vectors, and nucleic acids cannot completely exclude the risk of genomic integration. Proteins transduced via CPPs function transiently, but not stably in the cell. This kinetics could be suitable to mimic a differentiation process, because the expression of key transcription factors rapidly and dynamically fluctuates in defined periods in *in vivo* development and stable expression is rare. This method is useful as a means for directing the differentiation of iPS cells and for clinical application.

**Table 1:** Summary of pluripotent stem cell technologies via protein transduction methods.

CPPs	Proteins	Supplements	Technologies	Cell Types	References
Poly-arginine	OSKM	NA	Reprogramming	MEFs	[70]
Poly-arginine	OSKM	NA	Reprogramming	HNFs	[71]
NA	ES cell-derived extract proteins	Streptolysin O	Reprogramming	Mouse cardiac fibroblasts	[72]
Hydrophobic MTDs	OSKMN or OSKML	NA	Partial reprogramming	HDFs	[73]
TAT	Cre	NA	Recombination	Mouse ES cells	[60]
TAT	Cre	NA	Recombination	Human ES cells	[61]
TAT	FLP	dTAT-HA2 peptide	Recombination	Mouse or human ES cells	[62]
Poly-arginine	Cas9 and sgRNA	NA	Gene disruption	Human ES cells	[63]
NA	Cre or Cas9	Hypertonic solution and NDSB-201	Gene editing	Mouse or human ES cells	[22]
NA	Cre, TALE or Cas9	Anionic proteins and cationic lipids	Gene editing	Mouse ES cells	[23]
PTDs or Poly-arginine	Pdx1, NeuroD and MafA	NA	Pancreatic differentiation	Mouse ES cells or human iPS cells	[45]
TAT	Nkx2.2	NA	Neural differentiation	Mouse NSCs	[55]
TAT	Pax6	NA	Neural differentiation	Rat NSCs	[57]

CPP, cell-penetrating peptide; ES, embryonic stem; HDF, human dermal fibroblasts; HNF, human newborn fibroblast; iPS, induced pluripotent stem; MEF, mouse embryonic fibroblast; MTD, macromolecule transduction domain; NA, not applicable; NSC, neural stem cells; OSKM, Oct-4, Sox2, Klf4, c-Myc; OSKMN, Oct-4, Sox2, Klf4, c-Myc, Nanog; OSKML, Oct-4, Sox2, Klf4, c-Myc, Lin28; PTD, protein transduction domain; sgRNA, single-guide RNA; TAT, trans-activator of transcription.
